# APS8, a Polymeric Alkylpyridinium Salt Blocks α7 nAChR and Induces Apoptosis in Non-Small Cell Lung Carcinoma

**DOI:** 10.3390/md11072574

**Published:** 2013-07-16

**Authors:** Ana Zovko, Kristina Viktorsson, Rolf Lewensohn, Katja Kološa, Metka Filipič, Hong Xing, William R. Kem, Laura Paleari, Tom Turk

**Affiliations:** 1 Department of Biology, Biotechnical Faculty, University of Ljubljana, 1000 Ljubljana, Slovenia; E-Mail: ana.zovko@ki.se; 2 Department of Oncology and Pathology, Karolinska Biomics Center, Karolinska Institutet, 17176 Stockholm, Sweden; E-Mails: kristina.viktorsson@ki.se (K.V.); rolf.lewensohn@ki.se (R.L.); 3 Department of Genetic Toxicology and Cancer Biology, National Institute of Biology, 1000 Ljubljana, Slovenia; E-Mails: katja.kolosa@nib.si (K.K.); metka.filipic@nib.si (M.F.); 4 Department of Pharmacology and Therapeutics, College of Medicine, University of Florida, Gainesville, FL 32610, USA; E-Mails: hxing@ufl.edu (H.X.); wrkem@ufl.edu (W.R.K.); 5 Epidemiology, Biostatistics and Clinical Trials, IRCCS A.O.U. S. Martino-IST, National Institute for Cancer Research, 16132 Genoa, Italy; E-Mail: palearilaura@hotmail.com

**Keywords:** 3-alkylpyridinium polymers, apoptosis, nicotine, nicotinic acetylcholine receptor, non-small cell lung carcinoma

## Abstract

Naturally occurring 3-alkylpyridinium polymers (poly-APS) from the marine sponge *Reniera sarai*, consisting of monomers containing polar pyridinium and nonpolar alkyl chain moieties, have been demonstrated to exert a wide range of biological activities, including a selective cytotoxicity against non-small cell lung cancer (NSCLC) cells. APS8, an analog of poly-APS with defined alkyl chain length and molecular size, non-competitively inhibits α7 nicotinic acetylcholine receptors (nAChRs) at nanomolar concentrations that are too low to be acetylcholinesterase (AChE) inhibitory or generally cytotoxic. In the present study we show that APS8 inhibits NSCLC tumor cell growth and activates apoptotic pathways. APS8 was not toxic for normal lung fibroblasts. Furthermore, in NSCLC cells, APS8 reduced the adverse anti-apoptotic, proliferative effects of nicotine. Our results suggest that APS8 or similar compounds might be considered as lead compounds to develop antitumor therapeutic agents for at least certain types of lung cancer.

## 1. Introduction

Lung cancer (LC) is one of the most frequently diagnosed tumor types and is also the number one when it comes to death causing tumors [1]. With respect to LC histology, two major categories are small cell lung cancers (SCLC) and non-small cell lung cancers (NSCLC); the latter is further subdivided into squamous cell carcinomas, adenocarcinomas, and large cell carcinomas [2]. NSCLC, SCLC as well as normal lung cells have been shown to express various cholinergic signaling system molecules including acetylcholine, the vesicular acetylcholine ACh transporter, and nicotinic (nAChRs) and muscarinic (mAChR) receptors [3,4,5,6].

Nicotine, the main addictive component of tobacco, as well as some of its metabolites contributes to cancer development [7]. The inhalation of tobacco smoke containing nicotine and other compounds is associated with 90% of SCLC and 60% of NSCLC cases [8]. Although nicotine has not been shown to be carcinogenic, nitrosamine metabolites of nicotine are carcinogenic and it has been estimated that 2–3 DNA mutations result from every cigarette smoked [9]. Nicotine binds to various subtypes of nicotinic acetylcholine receptors (nAChRs) that are expressed on neurons and some non-neuronal cells [10,11,12,13,14]. It has been shown that certain tumor cells express an elevated number of nAChRs [6,12]. The homomeric α7 nAChR subtype seems to be particularly abundant in various tumor cells of the respiratory tract. It has been reported that exposure of lung adenocarcinoma cells to nicotine or ACh enhances expression of α7 nAChRs [15]. Activation of α7 nAChRs leads to elevated intracellular calcium levels sufficient to activate signal transduction pathways that prevent apoptosis and contribute to proliferation of LC cells [7,8,16,17]. Therefore, it has been suggested that α7 nAChR antagonists may be useful as therapeutics for suppressing rapidly proliferating tumor cells including LC cells [18,19,20].

Marine organisms have long been a source of antitumor compounds [21,22,23]. A polymeric mixture of 3-alkylpyridinium salts (poly-APS) which display a broad spectrum of biological activities was isolated from a marine sponge, *Reniera sarai*, which has been recently renamed to *Haliclona* (*Rhizoneira*) *sarai* (Pulitzer-Finali, 1969) [24,25,26,27]. Relevant to the present study, the most salient poly-APS effects are those that are preferentially toxic to NSCLCs [28].

**Figure 1:**
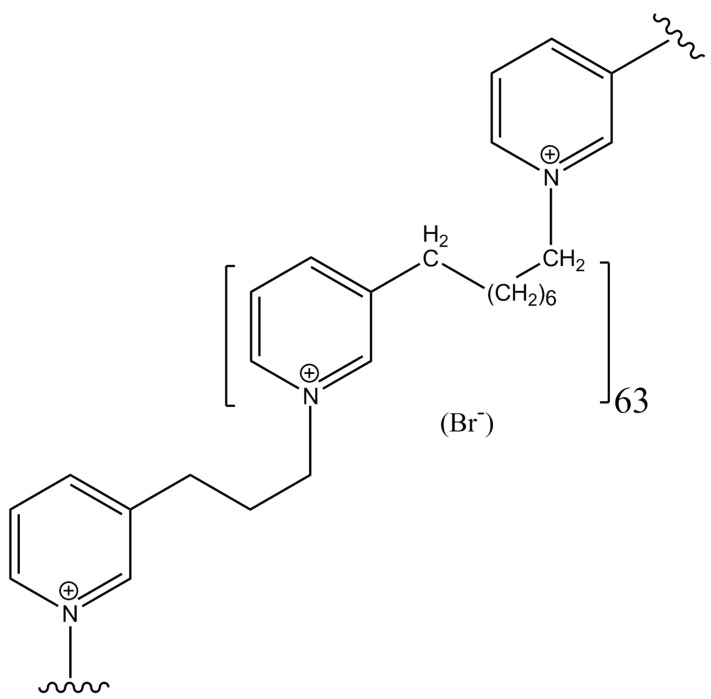
Chemical structure of APS8, the synthetic alkylpyridinium polymer, used in this study. Central monomeric unit linked to two neighboring units is shown. MW of the entire polymer is 11.9 kDa.

In view of the potential utility of poly-APS like compounds as anti-cancer agents, a series of synthetic analogs were prepared [29]. Preliminary results, demonstrated in this paper, revealed that at least one of these compounds, the 11.9 kDa analog APS8 (Figure 1), is a very potent α7 nAChR antagonist. Moreover, toxicity analysis in mice showed that APS12-2, a similar analog, was only moderately toxic in mice with an i.v. LD_50_ of 11.5 mg/kg [30], and the toxicity of APS8 used in this study is only slightly higher, about 8 mg/kg [31].

## 2. Results and Discussion

### 2.1. APS8 Induces Cytotoxicity in Lung Cancer (LC) Cells

**Figure 2:**
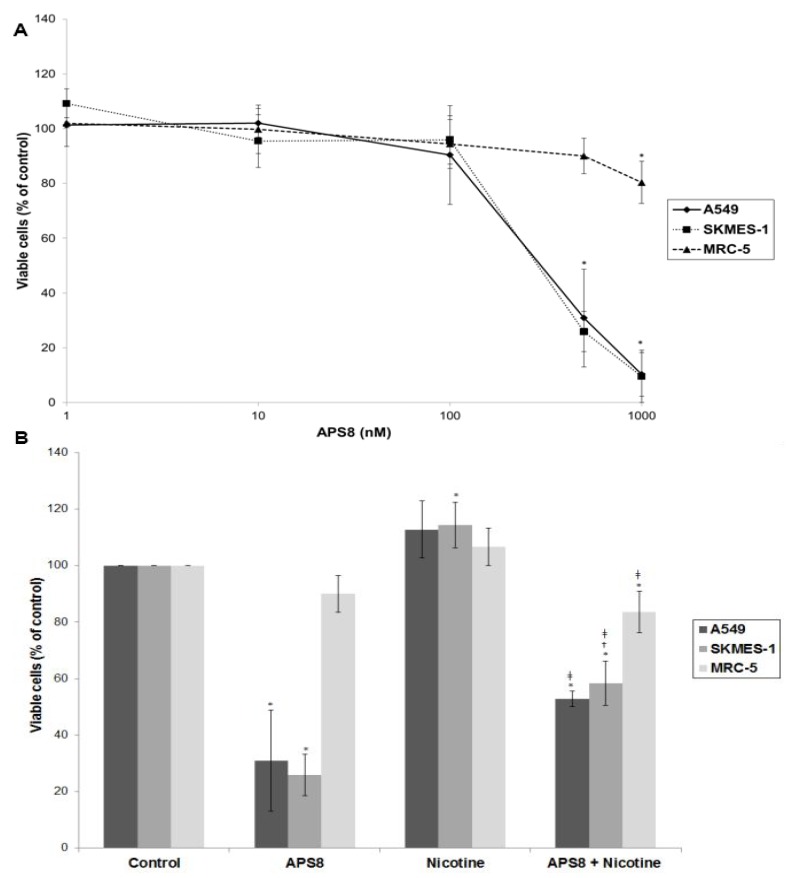
Viability of NSCLC (A549, SKMES-1) and normal lung fibroblast MRC-5 cells. (**A**) Viability of A549, SKMES-1 and MRC-5 cells treated with 0, 1, 10, 100, 500, and 1000 nM APS8 for 48 h was assessed by MTT assay. Each point represents the mean value of three independent experiments ± SE. Statistical analysis was performed by Student’s *t*-test. * *P* < 0.05; (**B**) Viability of A549, SKMES-1 and MRC-5 cells treated with APS8 (500 nM), nicotine (1 μM) or a combination of both compounds for 48 h. The MTT assay was used. Each point represents the mean value of three independent experiments ± SE. Statistical analysis was performed by ANOVA/Tukey-Kramer multiple comparison. * *P* < 0.05, compared with control; ^†^* P* < 0.05, compared with APS8 treatment; ^‡^* P* < 0.05, compared with nicotine treatment.

In order to examine if APS8 is cytotoxic to LC cells the SKMES-1 and A549 cell lines were treated with various concentrations of APS8 for 48 h and analyzed for cell viability by MTT-assay (Figure 2A). The effect on normal lung fibroblasts was also examined. APS8 in a concentration dependent manner strongly decreased viability of LC cell lines (IC_50_ 375 ± 4.89 nM for A549 cells and 362 ± 9.29 nM for SKMES-1 cells). Lung fibroblast cell line MRC-5 was largely unaffected thus incubation of these cells for 48 h with APS8 only resulted in a 20% decrease in cell viability at the highest concentration (1 μM). Next, the effect of APS8 on nicotine response was examined. Nicotine alone slightly enhanced cell survival of both A549 and SKMES-1 (13% for A549 and 14% for SKMES-1) (*p* < 0.05) while only a minor effect was observed with MRC-5 normal fibroblasts (6%) (Figure 2B). Importantly, APS8 significantly counteracted nicotine-induced effects in both LC cells (about 50%) while MRC-5 normal cells were much less affected. As compared to the APS8 only treatment, a combination of APS8 with nicotine caused a statistically significant (*p* < 0.05) increase of viable SKMES-1 cells (for 28%) and statistically insignificant increase of viable A549 cells (for 22%), while normal cells were not affected.

**Figure 3:**
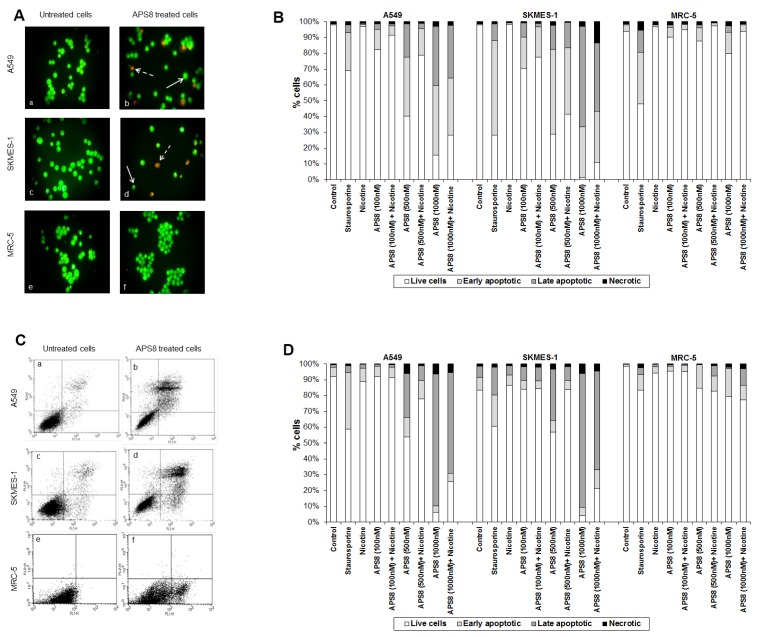
APS8 induces apoptosis in NSCLC but not in normal fibroblasts. (**A**) Apoptosis after APS8 treatment (500 nM, 48 h) in A549, SKMES-1, and MRC-5 were assessed by staining with acridine orange and ethidium bromide and analysis by fluorescence microscope. Photos were taken at 400× magnification. Dashed arrows indicate cells in early apoptosis and full arrows point to late apoptotic cells. Green cells are alive; (**B**) Induction of apoptosis in A549, SKMES-1, and MRC-5 lines as measured by dual staining. Cells were treated with staurosporine (2 μM), APS8 (100 nM, 500 nM, and 1000 nM), nicotine (1 μM) or combination of APS8 and nicotine. The graph indicates the percentage of cells in the single cell populations. Each point is the mean of three independent experiments. The protective effect of nicotine was significant only for A549 cancer cells treated with 500 nM of APS8 (* P < 0.05); (**C**) APS8 induction of apoptosis in A549, SKMES-1, and MRC-5 cell lines was measured by flow cytometric analysis of annexin V and propidium iodide stained cells at 48 h. Controls (a, c, and e) and APS8 (500 nM) treated cells (b, d, and f); (**D**) Induction of apoptosis in A549, SKMES-1 and MRC-5 lines by flow cytometry. Cells were treated with staurosporine (2 μM), APS8 (100 nM, 500 nM, and 1000 nM), nicotine (1 μM) or combination of APS8 and nicotine. The graph indicates the percentage of gated cells in each cell population. Each point is the mean of three independent experiments.

APS8 caused a prominent induction of apoptotic cell morphology in both A549 and SKMES-1 LC cells (Figure 3A, panel b and d). Quantification of APS8-induced apoptosis revealed a statistically significant (*p* < 0.05) and comparable response in A549 and SKMES-1 cells where about 40% of cells were found to be apoptotic after exposure to 500 nM of APS8 for 48 h (Figure 3B). Importantly, no induction of apoptosis was seen in normal fibroblasts MRC-5, which displayed the same nuclear morphology in the presence or absence of APS8 (Figure 3A, panel f and Figure 3B), thus corroborating a cancer cell specific apoptotic effect of APS8. The positive control staurosporine induced apoptosis in all cell types examined with the A549 cell line being least affected with only a 30% induction of apoptosis.

Next, we investigated whether APS8 is able to induce apoptosis in nicotine treated LC and fibroblasts (Figure 3B). As expected, nicotine alone did not trigger an apoptotic response in any of the cell types examined. LC cells treated with a combination of nicotine and APS8 displayed a greater resistance to apoptosis as compared to those treated only with APS8. Moreover, a greater sensitization was observed in SKMES-1 cells relative to A549 cells. In MRC-5 cells only the highest dose of APS8 induced limited apoptosis and this was reduced by the simultaneous exposure to nicotine.

The apoptotic properties of APS8 were also examined using annexin-V/PI staining. Exposure of A549 or SKMES-1 cells to APS8 resulted in typical apoptotic cells, evident as a shift to the right quadrants of the flow diagram (Figure 3C, panels b and d). Quantification of cell populations demonstrated a concentration dependent induction of apoptosis in both A549 and SKMES-1 cells (Figure 3D). Importantly, no induction of annexin-V was observed in normal MRC-5 fibroblasts (Figure 3C, panel f). Even at the highest concentration of APS8 used (1 μM), 80% of MRC-5 cells remained non-apoptotic (Figure 3D).

We also analyzed whether nicotine attenuates APS8 induced apoptosis using this assay (Figure 3D). Although nicotine slightly reduced APS8-induced apoptosis in A549 cells, apoptosis was still evident. A minor protective effect of nicotine was also evident in SKMES-1 cells. APS8 in any of the concentrations used did not induce apoptosis in MRC-5 fibroblasts or influenced their response to nicotine. Hence, our results support that APS8 has capacity to trigger an apoptotic response in LC cells whereas normal cells remain unaffected.

**Figure 4:**
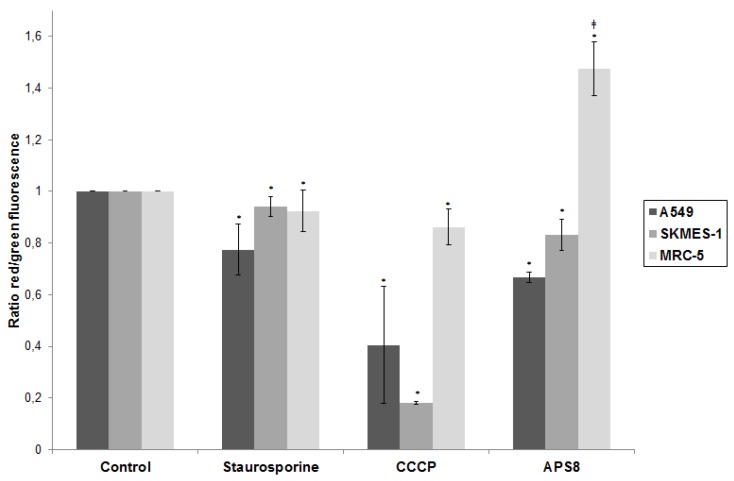
APS8 causes depolarization of mitochondria in NSCLC cells but not in normal fibroblasts. Ratio of red and green fluorescence following exposure to staurosporine (1 μM), CCCP (50 mM), APS8 (500 nM) for 48 h. Each point is the mean of three independent experiments ± SE. Statistical analysis was performed by ANOVA/Tukey-Kramer multiple comparison. * *P* < 0.05, compared with control; ^‡^* P* < 0.05, compared with staurosporine treatment.

### 2.2. APS8 Induces Mitochondrial Depolarization in LC Cells

We analyzed if APS8 could cause depolarization of mitochondria and in this way execute an apoptotic effect (Figure 4). First, we confirmed that the system is capable of detecting alterations in mitochondrial potential as illustrated by the prominent decrease in red/green fluorescence ratio in LC cells (60% for A549 and 80% for SKMES-1) after CCCP treatment. A substantial decrease of red/green fluorescence ratio was observed following APS8 treatment of A549 cells (about 35%) and SKMES-1 cells (about 15%) illustrating that APS8 at least in part works via mitochondrial depolarization. For comparison the staurosporine, an initiator of apoptosis, causes a 23% and 6% decrease in A549 and SKMES-1 cells respectively. There was no statistically significant difference between staurosporine and APS8 treated LC cell lines with respect to a decrease in red/green fluorescence ratio. In contrast, APS8 induced an increase in the red/green fluorescence ratio in MRC-5 cells, suggesting that APS8 does not cause mitochondrial permeabilization in normal fibroblasts (Figure 4).

### 2.3. APS8 Treatment Results in Increased Expression of Pro-Apoptotic Proteins and Down-Regulation of Anti-Apoptotic Proteins in LC Cells

**Figure 5:**
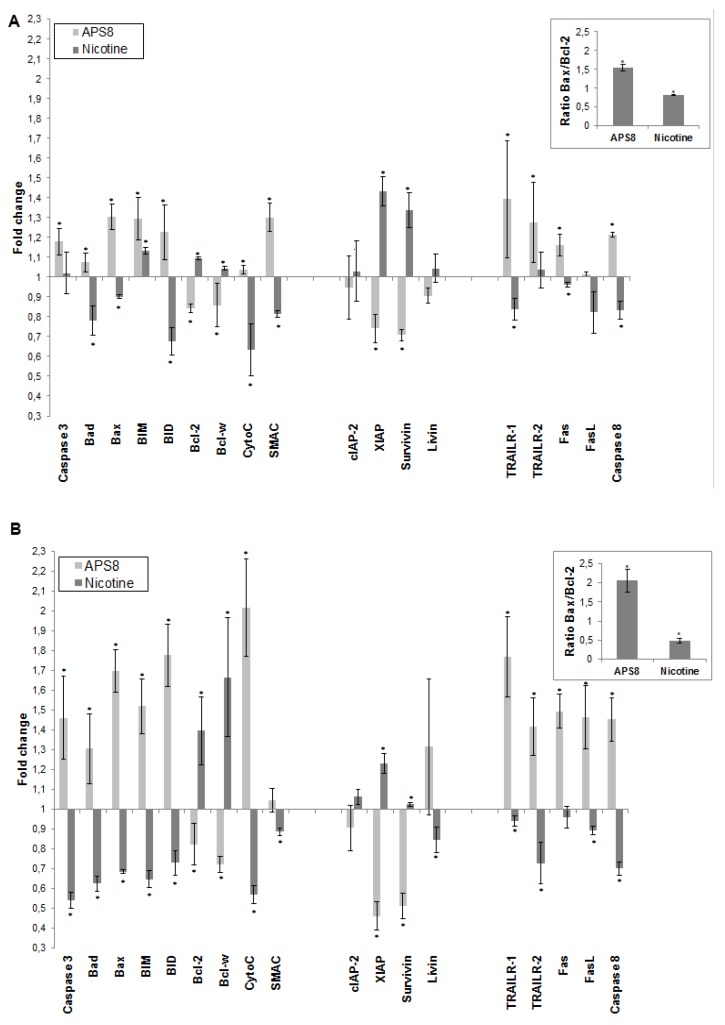
APS8 increases the expression of pro-apoptotic proteins and represses the expression of anti-apoptotic proteins. The change in expression of anti- and pro-apoptotic proteins was examined in A549 (**A**) and SKMES-1 (**B**) cells after treatment with APS8 (500 nM) or nicotine (1 μM) for 48 h using Human Apoptosis Antibody Array. Fold values relative to untreated cells are given. For presentation proteins are divided into three groups: proteins belonging to the intrinsic pathway, inhibitors of apoptosis (IAPs), and proteins belonging to the extrinsic pathway. Each point is the mean of three independent experiments ± SE. * *P* < 0.05 compared with control. The insert shows the ratio of main pro-apoptotic and main pro-survival proteins in APS8 and nicotine treated cells.

To further understand how APS8 influenced the apoptotic propensity of LC cells we examined the effect of APS8 on the expression of a number of pro-and anti-apoptotic proteins using the Human Apoptosis Antibody Array. APS8 (500 nM) treatment of A549 cells for 48 h resulted in up-regulation of several pro-apoptotic proteins, *i.e.*, bad, bax, bim and bid, cytochrome C, SMAC, TRAIL-R1 and TRAIL-R2, Fas, FasL, caspase-8, and caspase-3 albeit to the different extent (Figure 5A). Moreover, the majority of anti-apoptotic proteins, including bcl-2 and bcl-W, c-IAP-2, XIAP, survivin, and livin were down-regulated in A549 cells after APS8 exposure (Figure 5A).

Similarly, treatment of SKMES-1 cells with APS8 (500 nM) also resulted in an increased expression of the pro-apoptotic proteins, *i.e.*, bad, bax, bim and bid, cytochrome C, SMAC, TRAIL-R1 and TRAIL-R2, Fas, caspase-8 and caspase-3, with a slighter higher expression than was observed in A549 cells (Figure 5B). In accordance with results in A549 cells also SKMES-1 cells responded to APS8 with a down-regulated expression of the anti-apoptotic proteins bcl-2, bcl-W, cIAP-2, and XIAP (Figure 5B). Importantly, treatment of normal MRC-5 fibroblasts did not show any significant change in the expression of any of the proteins known to be involved in apoptosis (data not shown).

In addition, we examined how nicotine treatment of A549 and SKMES-1 cells influenced the expression of these pro- and anti-apoptotic proteins (Figure 5A,B). A majority of pro-apoptotic proteins, including bad, bax, bid, cytochrome C, SMAC, TRAIL-R1, Fas, FasL and caspase-8, were significantly down-regulated on nicotine exposure. Moreover, an increased expression of many proteins involved in signaling pathways known to prevent apoptosis, including bcl-2, bcl-W, cIAP-2, XIAP, surviving, and livin was evident. This was further illustrated when the bax/bcl-2 ratio was compared in ASP8 and nicotine treated cells. bax/bcl-2 ratio increased after APS8 treatment in both cell lines while it decreased after nicotine treatment (Figure 5A,B, inserts). These results further emphasize the selective activation of pro-apoptotic signaling in LC cells by APS8.

### 2.4. APS8 Treatment of Cancer Cell Lines Results in Activation of Caspase-9

**Figure 6:**
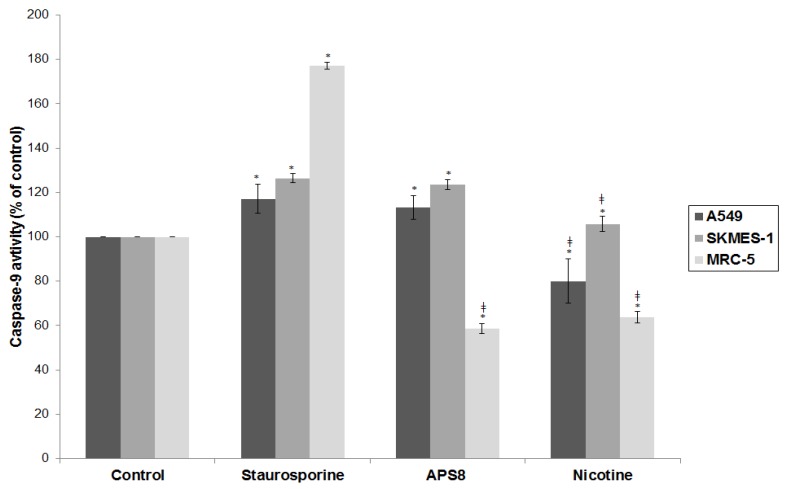
APS8 activates the intrinsic apoptotic pathway in NSCLC cells but not in normal fibroblasts. Caspase-9 activities relative to untreated control after treatment with staurosporine (2 μM), APS8 (500 nM), and nicotine (1 μM) for 48 h in A549, SKMES-1 or MRC-5 cells are given. Each point is the mean of three independent experiments ± SE. Statistical analysis was performed by ANOVA/Tukey-Kramer multiple comparison. * *P* < 0.05, compared with control; ^‡^* P* < 0.05, compared with staurosporine treatment.

Next, we examined caspase-9 activity, an effector molecule downstream of mitochondrial depolarization. Treatment of A549 and SKMES-1 with APS8 induced activation of caspase-9 to the similar extent as observed in positive control with staurosporine (*p* < 0.05). Nicotine treatment reduced the level of caspase-9 activity in A549 cells. In MRC-5 cells caspase-9 remained inactive after the treatment with APS8 or nicotine (*p* < 0.05) (Figure 6). These findings are in concordance with APS8 being a pro-apoptotic signaling molecule in LC cells, but not in normal fibroblasts.

### 2.5. APS8 is a Negative Regulator of Human α7 nAChRs

**Figure 7:**
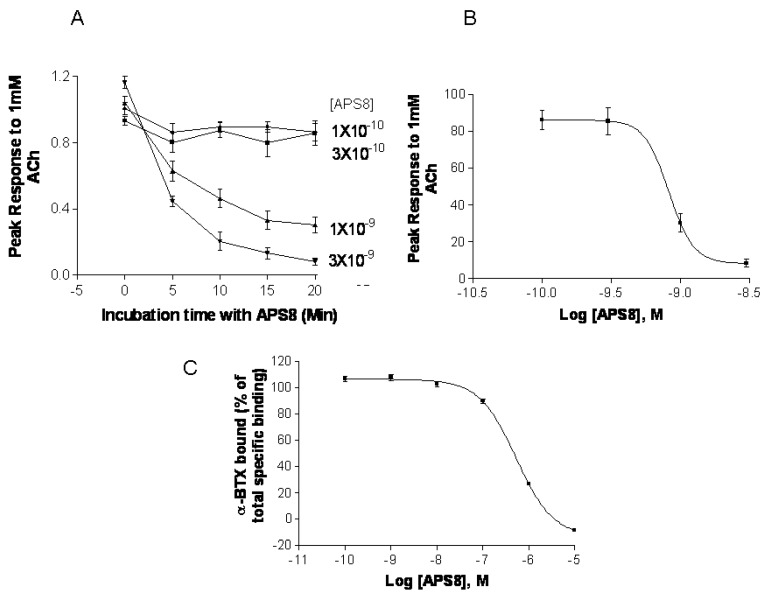
APS8 inhibits the response of human α7 nAChRs expressed in the *Xenopus* oocyte to a near maximally activating concentration of ACh (points represent mean values for five oocytes). In part (**A**) the time course of inhibition is shown; in part (**B**) the concentration dependence of APS8 inhibition at 20 min incubation is shown; Part (**C**) shows APS8 inhibition of the specific binding of [^125^I]-α-bungarotoxin to human α7 nAChRs expressed in cultured SHEP-1 cells. Each point is a mean value for four replicates.

In order to confirm that APS8 indeed is negative regulator of α7 nAChRs we examined its capacity to inhibit α7 nAChR activity *in vitro* systems, *i.e.*, *Xenopus* oocytes and SHEP-1 cells expressing human α7 nAChR (Figure 7). APS8 potently inhibited the responses of *Xenopus* oocytes expressing human α7 nAChR in a time dependent manner (Figure 7A,B). The IC_50_ was approximately 1 nM. The action of APS8 could at least partially be slowly reversed by prolonged washing of the oocyte with frog Ringer solution (results not shown). APS8 was also able to inhibit the specific binding of the α7 nAChR antagonist ^125^I-α-BTX in SHEP-1 cells system, but only at concentrations which were at least two orders of magnitude higher than was required to functionally inhibit α7 nAChR when expressed in *Xenopus* oocytes (Figure 7C). In summary, these results demonstrate that APS8 indeed is an antagonist of α7 nAChRs.

In the current study we analyzed the potential of the *Reniera sarai* derived synthetic compound APS8 as a LC antitumor agent. We show that it holds potential to selectively trigger a cytotoxic and pro-apoptotic response in NSCLC cells whereas normal fibroblasts remained unaffected. Moreover, we show that APS8 is an antagonist of the α7 nAChRs.

A variety of mAChR and nAChR subtypes are found on normal cells [3,14] but have an altered expression pattern in normal bronchial epithelial cells and airway fibroblasts of smokers, ex-smokers, and non-smokers [15]. Importantly, many cancer cell lines and primary cancer cells show a higher expression of mAChR and nAChR subtypes. Why LC cells have an increased expression of mAChR and nAChR is not completely clear but it might reflect adaption of the tissue to nicotine and other compounds inhaled by tobacco smoke [15]. Hence, α7 nAChRs might be a therapeutic target and in light of this our findings on the selective cytotoxic effects of APS8 to LC cells but not to normal lung fibroblasts are interesting.

Cytotoxicity for lung adenocarcinoma or squamous carcinoma NSCLC cell lines and the resistance of MRC-5 cells to natural poly-APS could be explained by the lack of nAChRs on the MRC-5 cells. Albeit, we did not evaluate the expression of α7 nAChRs in A549 or SKMES-1 cells, immunohistochemistry analyses of this receptor in A549 cells by the human protein atlas project demonstrated a high expression [32]. When preparing this manuscript, Lau *et al*., in fact, demonstrated by ELISA-based assay that A549 cells, as well as multiple other bronchioalveolar carcinoma (BAC) cell lines, do express α7 nAChRs [33]. Although our results reveal a therapeutic window for APS8, siRNA experiment towards α7 nAChRs would be required in order to confirm that these receptors are the specific APS8 target.

The ability to resist apoptosis is a hallmark of almost all types of tumor cells, including NSCLC, and this hampers the efficacy of chemotherapeutics [34]. ACh and the nAChR selective agonist nicotine have been found to protect NSCLC, SCLC, and some other tumor cells expressing α7 nAChRs against apoptosis induced by chemotherapeutic agents [16,35,36,37]. How the anti-apoptotic effect in NSCLC is achieved by ACh, other nAChR agonists, and the two main carcinogenic metabolites of nicotine *N*-nitrosonornicotine (NNN) and (4-(methylnitrosamino)-1-(3-pyridyl)-1-butanone (NNK), was recently revealed by Schuller [7]. In smokers, nicotine and its metabolites act predominantly through the activation of Ca^2+^ permeable α7 nAChRs, possibly because the heteromeric nAChR subtypes are already desensitized by low concentrations of these compounds during smoking. The influx of Ca^2+^ and also Na^+^ ions provokes membrane depolarization, which in turn activates voltage-gated Ca^2+^ channels, leading to an additional influx of Ca^2+^ [7]. The increased intracellular concentration of calcium triggers signal-transduction cascades involved in the regulation of numerous cellular processes: proliferation, angiogenesis, migration, differentiation, and apoptosis [7,8,38,39,40]. Thus, the α7 nAChR appears to be a promising target for the therapy of NSCLC or at least BAC as recently was proposed. One possible therapeutical approach would be the use of α7 nAChR antagonists either alone or in combination with other chemotherapeutic drugs. Such a combination would enhance apoptosis but also impede proliferation and metastasis of tumor cells [1,20]. Indeed, snake venom polypeptide α-toxins, *i.e.*, α-cobratoxin and α-bungarotoxin, which are potent natural ACh antagonists of muscle and several other nAChR subtypes were recently proposed as possible candidates to achieve such a goal [18]. However, due to their blocking actions on neuromuscular nAChRs, these peptide toxins are also highly toxic and therefore could be problematic for clinical treatment of cancer patients. Interestingly, APS8 (i.v. LD_50_ is about 8 mg/kg in mice, [31]) is significantly less toxic than snake α-toxins and APS8 possesses a more stable chemical scaffold suggesting that this compound might be suitable as an *in vivo* probe to test the therapeutic potential of α7 nAChR antagonists.

We show here that APS8 is an extremely potent α7 nAChR antagonist. The response of human α7 nAChRs expressed in *Xenopus* oocytes to 1 mM ACh, that alone produces a near maximal response, was completely blocked by about 3 nM APS8 after 20 min of incubation. The recovery from this blockade was very slow, requiring several hours (not shown). APS8 was only able to inhibit the specific binding of the snake toxin α-bungarotoxin to human α7 nAChRs at concentrations over 100× the function-inhibiting concentrations. These results point to a noncompetitive mode of APS8 mediated inhibition and binding of APS8 outside the actual ACh binding sites on this nAChR subtype. 

We anticipated that APS8 *in vitro* would inhibit proliferation and trigger apoptosis in tumor cell lines, but not in normal fibroblasts. Our hypothesis was based on our earlier observation that the parental compound from which APS8 was derived, the natural poly-APSs from the marine sponge *Reniera sarai*, can induce apoptosis of NSCLC cells, yet being well tolerated by normal cells [28]. Exposure to APS8 induced apoptosis in both lung cancer cell lines while normal fibroblasts MRC-5 did not undergo any significant apoptosis. These results were consistent when using different methods and confirmed our hypothesis.

The cytotoxic effect is probably exerted by inducing apoptosis through the binding to α7 nAChRs, which are expressed to a greater extent by cancer than by most normal cells. As expected from previous studies in a number of laboratories, nicotine promoted cell survival of LC cells. Therefore, we expected that nicotine in combination with APS8 would reduce the APS8 cytotoxic effect on cancer cell lines. Our results show that nicotine in combination with APS8 had only moderate effect on survival of A549 cells, while slightly stronger protection was evident on SKMES-1 cells. These results are in accordance with a previous study that suggested that nicotine has higher influence on proliferation of squamous carcinomas than of adenocarcinomas [19]. Nicotine in combination with APS8 somewhat also protected LC cells from apoptosis suggesting antagonistic effect of APS8 to nicotine.

Mitochondrial permeabilization is one of the early stages of apoptosis, which leads to the release of several pro-apoptotic factors, including cytochrome c and SMAC, which in turn facilitate the activation of the apoptosome and downstream caspase-9 and caspase-3 activation. Our results show that APS8 treatment of A549 and SKMES-1 causes a mitochondrial depolarization, suggesting that APS8 in part can induce apoptosis via the apoptosome-mediated pathway. Indeed, after exposure to APS8 we observed an activation of caspase-9, a typical initiator caspase of the intrinsic apoptotic pathway. 

To further test this hypothesis we used the RayBio^®^ Human Apoptosis Antibody Array kit. Treatment of A549 and SKMES-1 cells with APS8 (500 nM) increased the expression of caspase-3 which is an effector caspase activated by both intrinsic and extrinsic apoptotic pathways. The intrinsic pathway is activated by cellular stress, involves disruption of mitochondria and is controlled by bcl-2 family proteins. Induction of apoptosis relies on the ratio between pro-apoptotic (bax, bak, bcl-Xs, bad, bid) and anti-apoptotic (bcl-2, bcl-XL, bcl-W, mcl-1, A1) members of the bcl-2 family. Upon intrinsic apoptotic signal BH3 only proteins (bid, bad, PUMA, and NOXA) interact with the bcl-2 family, leading to inhibition of anti-apoptotic proteins and activation of pro-apoptotic proteins. Bax and bak proteins insert into the outer mitochondrial membrane causing the release of cytochrome c, bim, and SMAC from mitochondria. Released proteins then induce initiator caspase-9 activation and activation of effector caspase-3 [41,42]. The ratio between the levels of bax and bcl-2 proteins, at least in part, thus determines whether apoptosis will be induced with an increase in the bax/bcl-2 ratio causing a switch towards mitochondrial membrane permeabilization and activation of apoptosis. We show that treatment of both A549 and SKMES-1 cells expressing α7 and some other nAChR subtypes with APS8 triggers an increase in the expression of pro-apoptotic proteins bax, bad, bim and bid with a concomitant decrease in the levels of anti-apoptotic proteins bcl-W and bcl-2. Hence the bax/bcl-2 is increased, which may contribute to the observed increased apoptotic response of LC cells upon APS8 exposure. The data also revealed that both cytochrome c and SMAC, which are released from mitochondria during apoptosis, have an increased expression after APS8 treatment further increasing a pro-apoptotic capacity of the NSCLC cells. In summary, these results imply that APS8 induces apoptosis through the intrinsic pathway.

Our results also show that nicotine up-regulates bcl-2 and down-regulates bax, lowering the bax/bcl-2 ratio, which would favor a reduced apoptotic propensity of the NSCLC cells. Indeed, it was earlier suggested that inactivation of bax may be an essential step in anti-apoptotic mechanism induced by nicotine [39]. Our data show strong up-regulation of bax and down regulation of bcl-2 by APS8, quite opposite effects as compared to nicotine, which suggests that APS8 may at least in part counteract the protective effect of nicotine by increasing the bax/bcl-2 ratio. This effect was more prominent in SKMES-1 than in the A549 cancer cell line.

Another large group of proteins involved in regulation of the apoptosome activity are inhibitors of apoptosis proteins (IAPs), a family of pro-survival proteins [43]. In our experiments, all tested IAPs (cIAP-2, livin, XIAP and survivin) with the sole exception of livin in SKMES-1 cells, were down-regulated after exposure to APS8, whereas an increased expression was observed after nicotine treatment. Moreover, as mentioned earlier, SMAC displayed an increased expression in APS8 treated cells. This protein is an endogenous IAP antagonist and binds to IAP proteins, preventing their binding and inactivation of caspases, and thus promoting cell death. As was shown earlier, nicotine up-regulates IAPs, particularly XIAP, and in this way enhances resistance to anti-cancer treatments [44]. Our results suggest that APS8 can circumvent nicotine-induced resistance to apoptosis by interfering with IAPs allowing proper apoptosome function and downstream caspase activation to take place.

The Fas, TNF-R1, TNF-R2, and TRAIL (TRAIL-R1, -R2, -R3, and -R4) cell membrane receptors are key molecules of the extrinsic pathway of apoptosis and are activated upon binding appropriate ligands (FasL, TNF, and TRAIL) resulting in downstream caspase-8 activation followed by effector caspases (3, 6, and 7) activation [45]. Caspase-8 activation can trigger cleavage and activation of the BH3 only protein bid, which in turn can interact with bcl-2 proteins in mitochondria, resulting in intrinsic apoptotic pathway signaling and thereby activating effector caspases [46]. In the present study we show that TRAIL-R1 and TRAIL-R2 expression are enhanced following APS8 treatment while a decreased expression was observed after nicotine treatment. Although APS8 markedly increased the expression levels of Fas in A549 cells, no significant changes of FasL were noted. In SKMES-1 cell line both Fas and FasL showed a marked increased expression following the APS8 treatment. On the contrary, nicotine in both cancer cell lines slightly decreased the levels of Fas/FasL and TRAIL receptors. These results suggest that APS8 can activate components of death receptor signaling which at least in part might be cell type specific but, nevertheless, are oppositely regulated by nicotine.

## 3. Experimental Section

### 3.1. Synthetic Poly-APS Analog and Cell Cultures

APS8 (Figure 1) was synthesized using a previously described microwave-assisted polymerization procedure. This water-soluble compound has a molecular weight of 11.9 kDa and is stable at room temperature [29]. For the current experiments, a stock concentration of 10 mg/mL APS8 in deionized water was kept at 4 °C and further diluted in cell culture media upon use. 

Human lung adenocarcinoma A549 [47] squamous carcinoma SKMES-1 [48], and lung fibroblast MRC-5 [49] cells were purchased from the European Collection of Cell Cultures (ECACC). A549 and MRC-5 cells were grown in Dulbecco’s Eagle’s medium (DMEM) (PAA, Austria), while SKMES-1 was grown in RPMI1640 (PAA) both supplemented with 10% fetal bovine serum (FBS), l-glutamine and penicillin/streptomycin (Sigma-Aldrich, Germany). All experiments were carried out at 37 °C in 5% CO2.

### 3.2. Cell Viability Assay

Cytotoxicity was determined by 3-(4,5-dimethylthiazol-2-yl)-2,5 diphenyltetrazolium bromide (MTT) assay, as previously described [50], with minor modifications. Briefly, A549 and SKMES-1 cells were seeded at a density of 2500 cells/well and MRC-5 3500 cells/well into 96-well microtiter plates in five replicates for each of the three sets of experiments. Experiments were carried out 24 h post seeding when cells were about 80% confluent, at which time the growth medium was replaced with fresh medium containing different concentrations of APS8, 1 μM nicotine, or combination of both followed by incubation for 24, 48, or 72 h. Values shown are the mean of three or five replicates in three independent experiments ± SE.

### 3.3. Apoptotic Morphology Assessment

Cell morphology and percentage of apoptotic cells were studied by staining cells with a combination of the fluorescent DNA-binding dyes acridine orange (AO) and ethidium bromide (EB) (Sigma-Aldrich). The method is based on differential staining, where AO stains nuclei green, while EB stains red, and in which EB is excluded from viable cells. That allows enumeration of four populations: live cells which have normal bright green nucleus, necrotic cells which have light orange nucleus, early apoptotic cells which stain green with condensed chromatin, and late apoptotic cells which have condensed and fragmented chromatin and a emit bright orange-red color.

For apoptotic morphology experiments A549, SKMES-1, and MRC-5 cells were seeded at a density of 10^5^ cells/mL for 24 h and then treated with various concentrations of APS8 and/or nicotine for 48 h. Staurosporine (Sigma-Aldrich) (2 μM, 6 h) was used as a reference compound for apoptosis induction. After harvesting cells by trypsinization and centrifugation (81,614× *g*, 2 min), cell pellets were resuspended in 60 μL of medium. 50 μL of the cell suspension was mixed with 2 μL of AO/EB (1:1 w/w) dye mixture containing 100 μg/mL of AO and 100 μg/mL of EB in PBS. Cells were visualized and counted under a fluorescence microscope (Nikon Eclipse E-800, Nikon, Japan) at 400× magnification according to their color and structure. Pictures were taken with a digital camera (Nikon Coolpix 995, Japan). The quantification data shown are from three independent experiments; each time 300 cells were examined.

### 3.4. Analysis of Apoptosis by Annexin V and Propidium Iodide Staining

Apoptosis was also detected by staining cells with annexin V-FITC and propidium iodide (PI) (BD Pharmingen, San Diego, CA, USA). PI is a fluorescent dye that stains DNA. As the dye does not cross intact plasma membranes, only cells with a damaged plasma membrane that already are dead or in a late stage of apoptosis are stained. Annexin V-FITC is a fluorescent dye, which binds to phosphatidylserine (PS). During the early stages of apoptosis PS translocates from the inner to the outer layer of the plasma membrane and becomes available for binding to the annexin V-FITC. Thus, living cells do not stain, cells in early apoptosis are annexin V-FITC positive and PI negative, late apoptotic cells are both annexin V-FITC and PI positive and necrotic cells are annexin V-FITC negative and PI positive. For annexin-PI staining, cells (10^5^ cells/mL) were cultured for 24 h and then exposed to various concentrations of APS8 and/or nicotine for 48 h. Staurosporine (2 μM, 6 h) was used as a positive control. After treatment, cells were washed with PBS, trypsinized and resuspended in binding buffer and stained with 5 μL annexin V-FITC and 10 μL PI (50 μg/mL). The cells were incubated for 15 min at room temperature, in the dark. After incubation, 400 μL of binding buffer was added and cells were analyzed by flow cytometry (BD FACSCaliburTM, USA). Values shown are the mean of three independent experiments.

### 3.5. Mitochondrial Permeability

Increased mitochondrial permeability and release of pro-apoptotic proteins, e.g., cytochrome c to cytosol occurs prior to apoptosome activation. This transition is in part a result of activation and pore formation of the proapoptotic proteins (bax, bak) in the mitochondrial membrane. A specific dye, 5,5′,6,6′-tetrachloro-1,1′,3,3′-tetraethyl-benzimidazolylcarbocyanine iodide (JC-1), accumulates in mitochondria of normal cells, forming fluorescent aggregates emitting red light. When mitochondria are permeabilized the mitochondrial potential collapses and the dye is no longer concentrated within these organelles. In apoptotic cells JC-1 is dispersed throughout the cytosolic compartment in a monomeric form that emits green fluorescence. Therefore, the ratio between red and green signals indicates whether the mitochondrial potential of a cell is intact or not. To measure mitochondrial membrane potential, the Mitochondrial Permeability Transition Detection Kit JC-1 (ImmunoChemistry Technologies, Bloomington, MN, USA) was used according to the manufacturer’s instructions. Briefly, cells were plated at a density of 10^6^ cells/well and treated with 500 nM APS8 or 1 μM nicotine for 48 h. Staurosporine (2 μM, 6 h) and carbonylcyanide chlorophenylhydrazone (CCCP) (30 min) were used as a positive control for the induction of apoptosis and for the mitochondria depolarization, respectively. After, harvesting cells were resuspended in Mito PTTM JC-1 solution. After incubation for 15 min at 37 °C, cells were washed in assay buffer to remove any JC-1 excess and resuspended to achieve a cell suspension of 10^6^ cells/mL. For each test condition, 100 μL of cell suspension was dispensed into five replicate wells of a black 96 well microtiter plate. Cells were analyzed using a fluorescence plate reader using an excitation wavelength of 490 nm and emission wavelengths of 527 nm for green fluorescence and 590 nm for red fluorescence, respectively. Changes in mitochondrial potential were assessed by comparing the ratios of red (590 nm) and green (527 nm) fluorescence readings. A decrease of this ratio was accounted to depolarized mitochondria. Values shown are the mean of three independent experiments ± SE.

### 3.6. Expression of Pro-Apoptotic and Anti-Apoptotic Proteins

Expression of pro- and anti-apoptotic proteins in cells following APS8 or nicotine treatment was measured using a Human Apoptosis Antibody Array (RayBiotech Inc., USA). The Human Apoptosis Antibody Array kit simultaneously detects 43 apoptotic markers in cell lysates. Cells were seeded at a density of 10^6^ cells/mL and treated with 500 nM APS8 or 1 μM nicotine. Proteins were extracted using the cell lysis buffer contained within the kit. After lysis by freeze-thawing, and subsequent homogenization, lysates were centrifuged at 10,000× *g* for 5 min at 4 °C and supernatants were stored at −20 °C until further analyses. Protein concentration was determined by BCA Protein Assay Reagent (Pierce, USA). Protein array membranes were immersed in blocking buffer and incubated with 50 μg of proteins overnight at 4 °C. After washing with the kit buffers, samples were incubated overnight with biotinylated detecting antibodies at 4 °C and, after further washing, exposed overnight to Alexa Flour 555-conjugated streptavidin. Slides were completely dried by centrifugation (81,614× *g*, 3 min). Signals in the array membranes were depicted and quantified by a chemiluminescence imaging system with an Axon GenePix laser scanner, using the cy3 channel. For each protein signal, absorbance was determined using the Antibody Array Analysis Tool (Ray Biotech Inc.). Intensities of the individual spots were normalized to the internal positive control (standardized amounts of biotinylated IgG) printed directly onto the array. The expression of proteins was determined by comparing the intensities of the signals in untreated and treated cells. Only expression data on proteins that are directly involved in apoptosis and which showed statistically relevant increase after treatment (*p* < 0.05) are shown. Values shown are the mean of three independent experiments ± SE.

### 3.7. Caspase-9 Activity

A Caspase-9 Colorimetric Assay Kit (BioVision, USA) was used according to the manufacturer’s instructions. The assay is based on the binding of active caspase-9 to the sequence LEHD, which is labeled with a p-nitroanilide (pNA) chromophore. After cleavage from the labeled substrate, pNA absorbance can be quantified spectrophotometrically at 400 nm. Briefly, cells were plated at a density of 10^6^ cells/mL and treated with 500 nM APS8 or 1 μM nicotine for 48 h. Proteins were extracted from cells using cell lysis buffer within the kit, and the lysate was centrifuged at 10,000× *g* for 1 min and supernatant was stored at −20 °C. Protein concentration was determined with BCA protein assay. To 150 μg of total protein extract 50 μL of reaction buffer containing 10 mM DTT and 5 μL of 4 mM LEHD-pNA substrate were added. After 2 h incubation at 37 °C, pNA absorbance was quantified at 400 nm (Tecan Genios, Austria). Values shown are the mean of three independent experiments ± SE.

### 3.8. Effect of APS8 on *α*7 nAChRs

*Xenopus* oocyte functional experiments and radioligand binding experiments were performed in order to assess the effects of APS8 to α7 nAChR. Human α7 mRNA was expressed in *Xenopus* frog oocytes, as previously described [51]. Individual oocytes were placed in a 20 μL oocyte perfusion chamber (AutomateScientific, Berkeley, CA, USA) and perfused at room temperature in Frog Ringer’s solution (115 mM NaCl, 2.5 mM KCl, 10 mM HEPES, 1.8 mM CaCl2, pH 7.3) containing 1 μM atropine to block potential muscarinic responses. The two-microelectrode voltage-clamp technique was used to measure current responses at a constant holding potential (−60 mV). The electric resistance of the voltage-measuring microelectrode, filled with 3 M KCl solution, was 0.5–3.0 MΩ; the resistance of the current-passing electrode (containing 250 mM CsCl, 250 mM CsF, and 100 mM EGTA) was 0.5–2.0 MΩ. Membrane currents were recorded with an AxoClamp-2 (Axon Instruments, Union City, CA, USA). Sampling rates were between 5 and 10 Hz and the data were filtered at 10 Hz. Acetylcholine (ACh) was rapidly applied using a ValveLink8.2 controller (AutoMate Scientific, Berkeley, CA, USA). ACh applications were 1.0 s in duration, unless otherwise noted. The perfusion rate was 2.0 mL/min. Initially each oocyte received two control applications of a near maximal stimulatory concentration of 1 mM ACh to achieve a consistent response. Clampfit 8.1 (Axon Instruments, USA) was used for data acquisition. The concentration-response curve for APS8 inhibition of the 1 mM ACh response was fitted with a sigmoidal dose-response curve software (Prism 3.0, La Jolla, CA, USA) allowing a variable slope.

APS8 displacement of specific binding of ^125^I-α-bungarotoxin (α-BTX) to human α7 nAChRs expressed in SHEP-1 cells was measured according to methods essentially as previously described [51]. The membranes (50 μg per tube) were suspended in ice cold binding saline (120 mM NaCl, 5 mM KCl, 2 mM CaCl2, 1 mM MgCl2, 50 mM Tris-Tris-HCl buffer, pH 7.4). Measurements of α7 nAChR affinity were done by displacement of ^125^I-α-BTX (136 Ci/mmol) binding; these experiments required incubation for three hours at 37 °C to assure equilibration [50]. The cell membranes, ^125^I-α-BTX (final concentration ~0.2 nM) and APS8 were incubated together in 48 13 × 100 mm disposable glass culture tubes, each possessing a final volume of 0.5 mL. Nonspecific ^125^I-α-BTX binding was measured in the presence of a final concentration of 1 mM (S)-nicotine hydrogen tartrate salt (Sigma, St. Louis, MO, USA). After incubation, the radioligand bound membranes in each of the 48 tubes were simultaneously collected by vacuum filtration (Brandel cell harvester, Gaithersburg, MD, USA) on Whatman GF/C glass fiber filters pre-soaked in 0.5% polyethylenimine for 45 min to reduce nonspecific binding. The radiolabeled membranes were rapidly washed three times with 3 mL ice-cold binding saline to separate bound from free radioligand. Filters containing ^125^I-α-BTX bound membranes were placed in 4 mL vials and counted with a Beckman 5500B gamma counter (Fullerton, CA, USA). Displacement assay binding data were analyzed using GraphPad Prism software (San Diego, CA, USA). The mean counts per minute values for each concentration of APS8 were obtained from four replicates. The data were fitted to a sigmoidal concentration response curve from which the Hill slope (*n*) and IC_50_ (*X*) values were estimated:

*Y* = Bottom + (Top − Bottom)/(1 + 10^(*X*^^ − ^^log(IC50)*n*^)

Here, Top (top of the curve) is the maximal specific binding plateau of radioligand and Bottom (bottom of the curve) is the minimum specific binding plateau observed at high concentrations of the displacing ligand.

### 3.9. Statistical Analysis

All experiments were repeated three to five times. All data were expressed in terms of mean value and standard deviation. Statistical significance analysis was determined using Student’s *t*-test or analysis of variance (ANOVA) followed by Tukey-Kramer multiple comparison. *P* < 0.05 was considered significant.

## 4. Conclusions

Our data reveal that in the tested cancer cell lines APS8 triggers the intrinsic apoptotic pathway although there is some evidence that it might be also involved in apoptotic response via the extrinsic pathway. While further studies are needed, the present work suggests that compounds with molecular characteristics similar to APS8 might represent a new molecular approach for treating certain types of lung cancer.
